# A NMF based approach for integrating multiple data sources to predict HIV-1–human PPIs

**DOI:** 10.1186/s12859-016-0952-6

**Published:** 2016-03-08

**Authors:** Sumanta Ray, Sanghamitra Bandyopadhyay

**Affiliations:** Department of Computer Science and Engineering, Aliah University, Kolkata-700156, West Bengal, India; Machine Intelligence Unit, Indian Statistical Institute, Kolkata-700108, West Bengal, India

## Abstract

**Background:**

Predicting novel interactions between HIV-1 and human proteins contributes most promising area in HIV research. Prediction is generally guided by some classification and inference based methods using single biological source of information.

**Results:**

In this article we have proposed a novel framework to predict protein-protein interactions (PPIs) between HIV-1 and human proteins by integrating multiple biological sources of information through non negative matrix factorization (NMF). For this purpose, the multiple data sets are converted to biological networks, which are then utilized to predict modules. These modules are subsequently combined into meta-modules by using NMF based clustering method. The integrated meta-modules are used to predict novel interactions between HIV-1 and human proteins. We have analyzed the significant GO terms and KEGG pathways in which the human proteins of the meta-modules participate. Moreover, the topological properties of human proteins involved in the meta modules are investigated. We have also performed statistical significance test to evaluate the predictions.

**Conclusions:**

Here, we propose a novel approach based on integration of different biological data sources, for predicting PPIs between HIV-1 and human proteins. Here, the integration is achieved through non negative matrix factorization (NMF) technique. Most of the predicted interactions are found to be well supported by the existing literature in PUBMED. Moreover, human proteins in the predicted set emerge as ‘hubs’ and ‘bottlenecks’ in the analysis. Low p-value in the significance test also suggests that the predictions are statistically significant.

## Background

Interaction between proteins is considered to be an important biochemical reactions which controls different biological processes. Analysis and prediction of protein-protein interactions (PPIs) between viral and host proteins is an important step to uncover the underlying mechanism of viral infection in host cell machinery. Human Immunodeficiency Virus-1 (HIV-1) belongs to a special class of viruses called retrovirus, in which it is placed in the subgroup of lentiviruses. It consists of a single stranded RNA which encodes 19 proteins. HIV-1 virus relies on the human cellular machinery for its replication. It hijacks the human cellular mechanism and uses it to produce viral genetic material.

One of the most important parts of HIV research is to discover the underlying mechanism of interactions between HIV-1 protein and human protein. Predicting such interactions contributes a major task in PPI research for antiviral drug discovery as well as treatment optimization. There exist several approaches that exploit different methodologies for predicting HIV-1 –human PPIs (HHPPIs). These are approximately categorized into three groups: supervised classification based approach, structural similarity based approach and association rule mining based approach [1]. One of the first attempts is reported in [2]. Here a random forests classifier is trained using 35 features derived from different data sources. As an extension of this work [3] proposed a semi-supervised multi tasking approach to improve the predictive performance. Here modified feature set is used to precisely capture the HIV related information. In [4], a supervised classification technique based on Support Vector Machine (SVM) is proposed to predict HHPPIs. Here protein domains, sequence and PPI information are incorporated in the feature set. In [5] protein structure information available in Protein data bank (PDB) along with the experimentally verified PPI information are utilized for prediction. In [6] an association rule mining based approach is utilized. As an extension of this work, biclustering technique based on association rule mining is developed in [7] where the type and direction of interactions are also taken into account. Most of these works predominantly used the HIV-Human Protein Interaction Database (HHPID) [8] for prediction and validation.

**Fig. 1:**
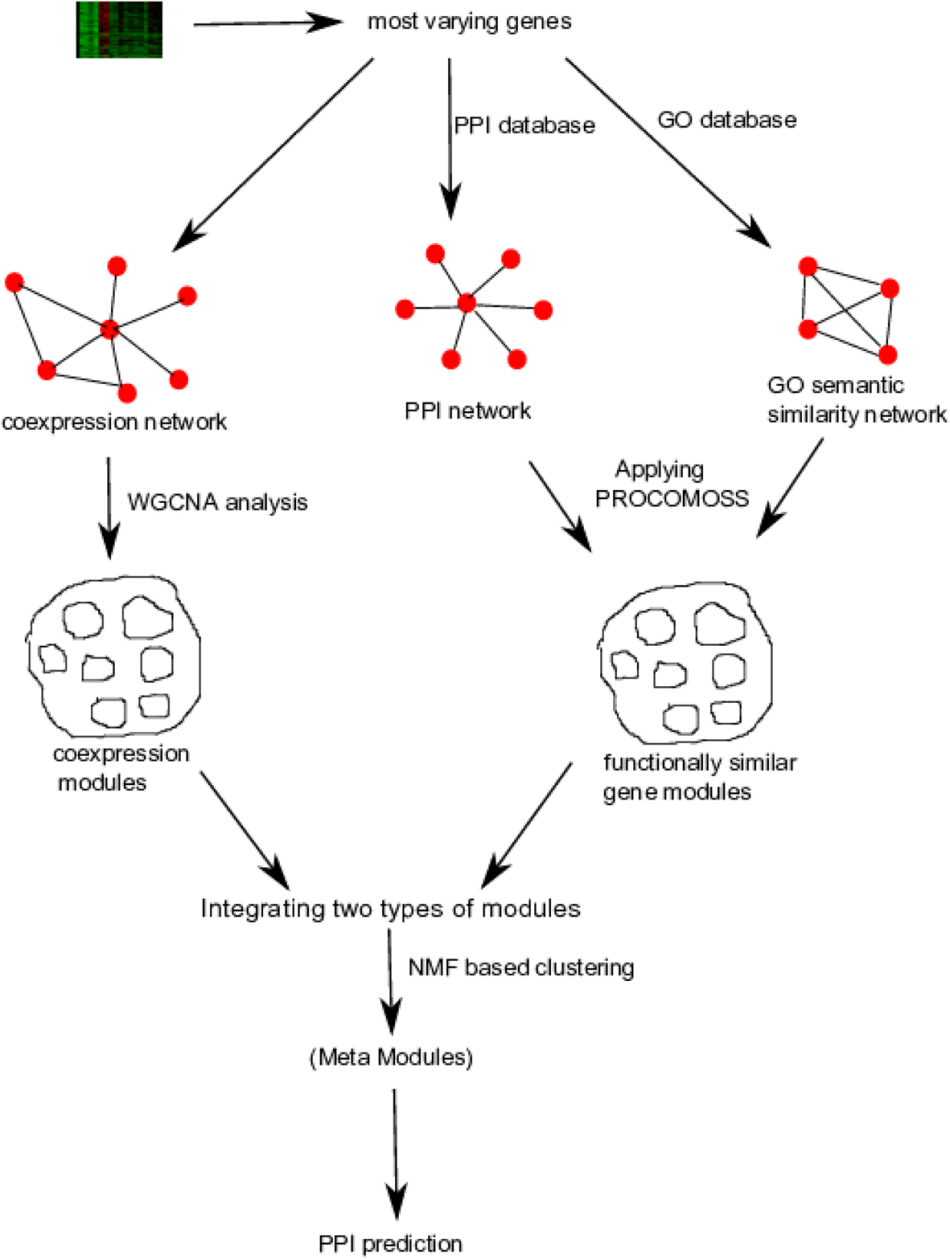
Overall summery of the proposed methodology

The above approaches utilize single data source for predicting HHPPIs. To the best of our knowledge, no study exists where different types of biological data sources are integrated for predicting HHPPIs, although such integration has already proved to be effective in other domains [9, 10]. In this work, we have proposed a framework where three sources of information, namely, gene expression, PPIs and Gene Ontology based similarity, are integrated through NMF based clustering. Meta modules are identified and subsequently these are used for predicting novel PPIs. For integration purpose all the data sources are first converted into respective biological network. For keeping the similar structure of all the data sources this conversion is necessary. Gene expression dataset is converted into coexpression network, while PPI information and Gene Ontology information are converted into PPI network and GO semantic similarity network, respectively. These networks are then subsequently utilized for detecting modules. For this purpose, we have utilized two popular module finding frameworks. Weighted Gene Coexpression Network Analysis (WGCNA) [11] is utilized for detecting coexpression modules, while Protein Complex detection using Semantic Semilarity (PROCOMOSS) [12] is utilized for detecting functionally coherent protein modules. The identified modules are then integrated through NMF based clustering method. The integrated meta-modules inherit the intrinsic properties of all the data sources and are regarded as consensus of these two categories of modules. We have observed that HIV-1 interacting proteins in meta modules show significantly high interactions among them. This information is used for prediction of HHPPIs. The overall process of our methodology is shown in Fig. 1.

## Methods

In this section we have discussed the proposed methodology for predicting PPIs between HIV-1 and human protein.

### Dataset preparation

Our study is based on three biological data sources, gene expression data, protein-protein interaction data and Gene Ontology data. We downloaded the gene expression dataset (GSE6740 series) from GEO database (http://www.ncbi.nlm.nih.gov/geo) which consists of stage specific expression value of CD4+ and CD8+ cells collected from a cohort of untreated HIV-1 infected individuals. From this, we have taken expression data of acute stage infection from which we select 2828 most varying genes among the 22,284 genes. We prepared a coexpression matrix of 2828 × 2828 dimension, where each entry in the matrix represents Pearson correlation similarity. For the PPI data, we downloaded human PPI dataset from HPRD [13]. From the whole human PPI dataset we take PPI information of the 2828 selected genes. The 1/0 entry in the adjacency matrix of this data represents presence/absence of PPI connection. Gene Ontology information of those selected genes are also collected from GO dataset. GO terms are indexed by Uniprot gene id, whereas the proteins in PPI dataset and in gene expression dataset are indexed by official gene symbol and affimetrix probesets respectively. To resolve the mapping between gene ids we have used a widely used gene id conversion tool named David Bioinformatics resource (https://david.ncifcrf.gov/home.jsp) [14]. We take the average expression value of multiple probs which match a gene id in HPRD dataset. Conversely, we take all the gene ids of HPRD dataset which match with one probe in affimetrix probeset. Similarly, we retain all uniprot ids in GO database for a particular gene symbol. Functional similarity between two proteins are measured by computing semantic similarity between GO-terms associated with these proteins. For computing semantic similarity we use biological process annotation of the GO terms. We have compiled a GO based semantic similarity network using the semantic similarity measure proposed in [15].

**Fig. 2:**
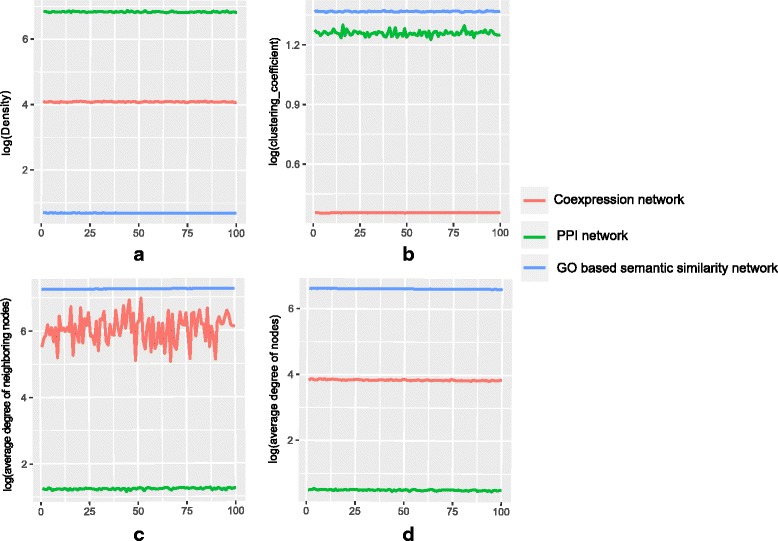
Figure shows the change of four topological metrics for randomly removal of nodes from the three networks viz., PPI network, coexpression network, and GO based semantic similarity network. For the three networks, change of four metrics: network density, average clustering coefficient, average degree of the network and average degree of the neighboring nodes are shown in panel (**a**), panel (**b**), panel (**c**) and in panel (**d**) respectively

To investigate the stability of these three compiled networks, we perform a perturbation experiment. For this purpose we randomly remove some portion of the networks and compute some topological metrics. We repeat this experiment 100 times and investigate whether the topological characteristics of the network change due to random removal of nodes. Here, density, average clustering coefficient, average degree of the network and average degree of the neighboring nodes of the network are measured. For coexpression network and GO based semantic similarity network weighted version of connectivity and clustering coefficient are used [11]. Figure 2 shows change of these topological properties for the networks. From this figure it is noticed that the networks are stable under the random removal of nodes.

### Network construction and module detection

**Fig. 3:**
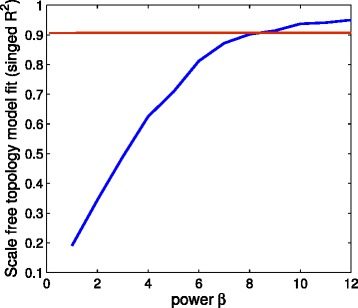
Scale-free topology fitting index (*R* ^2^) at different threshold value (*β*) At *β*=9 the metric $\frac {log(p(k))}{log(k)}$ converges to 1. The red line signifies the value of *β* for which the network obeys scale free property

We have followed WGCNA framework for detecting modules from coexpression network. WGCNA is utilized to find clusters or modules in gene coexpression network which follows scale-free topology criterion. For constructing coexpression network we have followed the methodology proposed in [11]. At first step, coexpression network is formed by computing Pearson correlation between each pair of gene expression profile. Here the following ‘scale free topology criterion’ is used for choosing an appropriate parameter to construct coexpression network: ‘choose the parameter for which the network satisfies scale free topology criterion at least approximately’. To achieve this, we have raised all correlation values to a power *β* and plot the ratio $\frac {log(p(k))}{log(k)}$ with respect to different *β* values, where *k* represent connectivity and *p*(*k*) is the probability of nodes having connectivity *k*. For every scale free network *log*(*p*(*k*)) and *log*(*k*) show linear relationship, so the value of $\frac {log(p(k))}{log(k)}$ converges to 1 when the corresponding network obeys scale free properties. Figure 3 shows at *β*=9 it converges to 1 approximately.

After constructing the coexpression network we have utilized a topological overlap (TOM) based dissimilarity measure to capture the relative connectedness for each pair of nodes. Modules are generally represented as a set of nodes with high topological overlap [16]. The topological overlap matrix corresponding to the network is converted to a dissimilarity matrix and then average linkage hierarchical clustering is performed to detect modules. The resulting modules are identified from the dendrogram by cutting it at a specific level using a dynamic tree cut algorithm [17]. Total 30 coexpression modules are identified.

For detecting functional homogeneous modules we have utilized PROCOMOSS [12] algorithm. PROCOMOSS is a multiobjective framework which cluster PPI network based on the semantic similarity information among gene ontology terms of proteins. First, we collect PPI information for the selected 2,828 proteins from the PPI dataset downloaded from HPRD [13]. Next, we construct a PPI network for these selected proteins using the PPI information. The constructed network are undirected and unweighted. To build semantic similarity network among the selected proteins, GO based semantic similarity measures is utilized here. Here, we have used a measure proposed in [15] for computing the semantic similarity between each pair of protein. The similarity value serves as the weight between each pair of nodes in the network. These two networks are used to find modules that captures both the PPI and gene ontology based information. The similarity between proteins are measured by applying GO based semantic similarity measures. By integrating these two information PROCOMOSS detects dense clusters in which proteins share similar functionality. We have applied this algorithm to detect modules in the PPI and GO similarity dataset. Total 40 modules are extracted from these data using PROCOMOSS. These two categories of modules are then integrated by using NMF based technique.

### Integration of modules using NMF based clustering

**Fig. 4:**
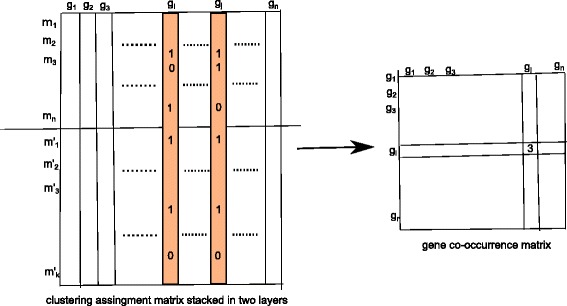
Integrating two categories of module into one matrix. The entry (*g*_*i*_,*g*_*j*_) in gene co-occurrence matrix is computed by performing the logical AND operation between two columns corresponding to *g*_*i*_ and *g*_*j*_ in the two layered clustering assingment matrix, and taking sum of this ANDing result

In the integration phase the two categories of identified modules are combined to preserve the contribution of all three original dataset in the newly formed groups by using NMF based clustering technique. First, individual clusters are formed by applying WGCNA and PROCOMOSS framework on the coexpression network and PPI and Gene semantic similarity network, respectively. Formally, we have constructed a set of representative clustering *M*={*M*_1_,*M*_2_}, one for each dataset. *M*_1_ consists of coexpressed modules whereas *M*_2_ are the functionally homogeneous modules predicted using PROCOMOSS. Each representative clustering or module set can be viewed by a non negative membership matrix as follows: $M_{i} \in R^{n \times k_{i}}\phantom {\dot {i}\!}$, where *n* is the number of proteins/genes and *k*_*i*_ represents number of clusters in module set *M*_*i*_. Transposing these two matrices and arranging them vertically, the resulting matrix can represented as *X*^*K*×*n*^ where *K*=*k*_1_+*k*_2_. Each row represents an individual cluster whereas column corresponds to a gene/protein. Each entry in the matrix corresponds to binary 0 or 1 which represents the membership of gene/protein in that cluster/module. Searching for ‘1’ columnwise, corresponds to searching the modules in which the gene/protein belongs. To investigate the gene/protein pair which simultaneously co-occurs in same modules we perform a logical AND operation on each pair of columns and make summation of this. This represents number of modules in which the gene/protein pairs co-occur. Following this conventions we compile another matrix which stores the information about the co-occurrence of pair of genes/proteins. Figure 4 shows the whole process. The resulting matrix stores the information about the co-occurrence of gene pairs in different category of modules. The primary goal of the integration process is to select a set of meta-modules consisting of genes/proteins which co-occur in two different categories of modules, thus preserving the characteristics of two different data resources in those meta modules. NMF based clustering is established to be a promising technique for multiple data clustering and consensus clustering [18]. The formulation of NMF can be extended to the clustering of nonnegative data. The general formulation of NMF takes the form: given a nonnegative matrix *X*∈*R*^*n*×*m*^ and a reduced rank *k*≤*n* the task is to provide a lower-rank matrix approximation as: *X*∼*VH*^*T*^, where *V,H*≥0. Here *V*∈*R*^*n*×*k*^ represents the projection of original data to a new set of basis vectors. This is also represented as meta cluster centroid, where k is the number of meta clusters. These metaclusters can be additively combined using the column of matrix *H*∈*R*^*k*×*m*^. For measuring the reconstruction error between original and the factors *V* and *H*, the Frobenious norm is utilized here.

### Interaction prediction

**Fig. 5:**
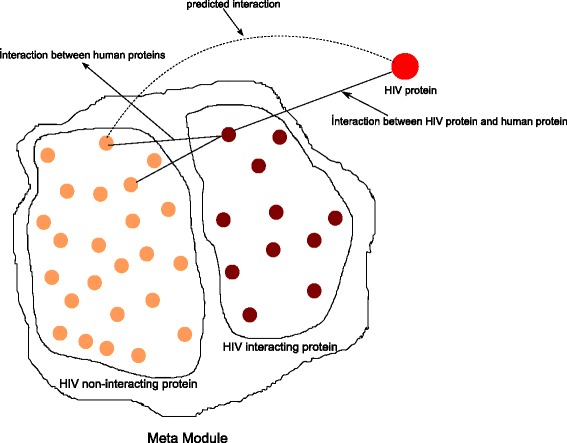
A toy example of identified meta-modules. It consists of HIV interacting and HIV non-interacting proteins. Among the non interacting set the prediction is performed

From the extracted meta-modules we predict some novel interactions by integrating HIV-human PPI dataset published in [8]. For this, we search the meta-modules for finding HIV-1 interacting proteins. Figure 5 shows the proposed technique for interaction prediction.

Consider a meta-module consisting of *n* proteins as *M*_*i*_=*g*_1_,*g*_2_,…*g*_*k*_,*g*_*k*+1_…*g*_*n*_, where first *k* proteins are interacted with HIV-1. As the rest of the proteins are members of the same module with the first *k* HIV-interacting proteins, so it is obvious that they share some similar characteristics with them. Here the similarity occurs in terms of PPI information, expression similarity information and gene ontology information.

The following mechanism is followed for predicting interactions:

The proteins in a meta module is divided in two subsets: HIV-1 interacting (*H*) and HIV-1 non-interacting (*G*). Protein *g*_*i*_∈*G* is predicted to interact with HIV-1 protein *h*_*j*_ if the following two conditions simultaneously hold:

- *g*_*i*_ shares same module with the *k* HIV-1 interacting proteins.
- *g*_*i*_ should be interacted with protein *g*_*k*_, where *g*_*k*_∈*H*, and *g*_*k*_ is interacted with HIV-1 protein *h*_*j*_

To prove the significance of the proposed mechanism we perform a statistical analysis. It is based on the following postulate:

*HIV interacting proteins in the identified meta-modules exhibit significantly high interactions among them.*

To prove the hypothesis, we first pick 40 random modules form HIV interacting human protein, retaining the size same as original meta-modules. We count the number of interactions among the HIV interacting proteins in the predicted meta modules as well as in the random modules by using STRING database. We find the number of interactions among the HIV interacting proteins in the meta-modules are significantly high (*p*-value =6.80e–05) by using the Wilcoxon Ranksum test.

### Predicted interactions are statistically significant

**Fig. 6:**
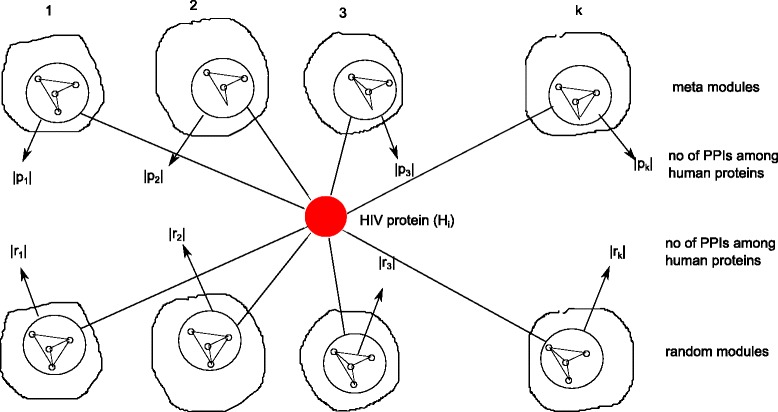
Statistical test to compare number of interactions among human proteins interacted with HIV-1 protein *H*_*i*_ in the predicted meta-modules

Figure 6 illustrate the statistical validation of the predicted interactions. one key assumption of our proposed method is that HIV interacted proteins within a meta module have significantly higher interactions. Here we investigate this in more detail for each HIV-1 protein. Let us assume *P*_*i*_=[*p*_1_,*p*_2_,…*p*_*k*_] represents number of interactions among human proteins in *k* meta modules for HIV-1 protein *H*_*i*_. We have compiled *k* meta modules randomly, retaining the size same as original, and count the interactions to form the set of interactions *R*_*i*_=[*r*_1_,*r*_2_,…*r*_*k*_] similar to *P*_*i*_, for each HIV-1 protein *H*_*i*_. We have utilized Mann-Whitney U test to determine number of interactions in the set *P*_*i*_ are significantly higher than the interactions in set *R*. The test produces p-value for each of the HIV-1 protein in the predicted interactions. We have shown this in Table 1. The predicted interactions consist of 17 HIV-1 proteins, among them the p-value of env_gp120 and Tat is significantly lower.

**Table 1:** *p*-value obtained from Mann Whitney U test for each HIV protein

HIV	Capsid	env_	env_	env_	integrase	matrix	Nef	nucleocapsid	retropepsin	Rev	Tat	Vif	Vpr	Vpu	Pol	Gag_pr55
protein		gp120	gp160	gp41												
name																
*p*_value	0.0031	0.00091	0.02608	0.00903	0.0156	0.0183	0.082	0.0174	0.0071	0.0115	0.00030	0.0515	0.0200	0.1817	0.1606	0.0021

## Results and discussions

In this section we describe the results of our proposed method for predicting interactions between HIV-1 and human proteins.

### Degree and betweenness centrality of proteins in identified meta-modules

**Fig. 7:**
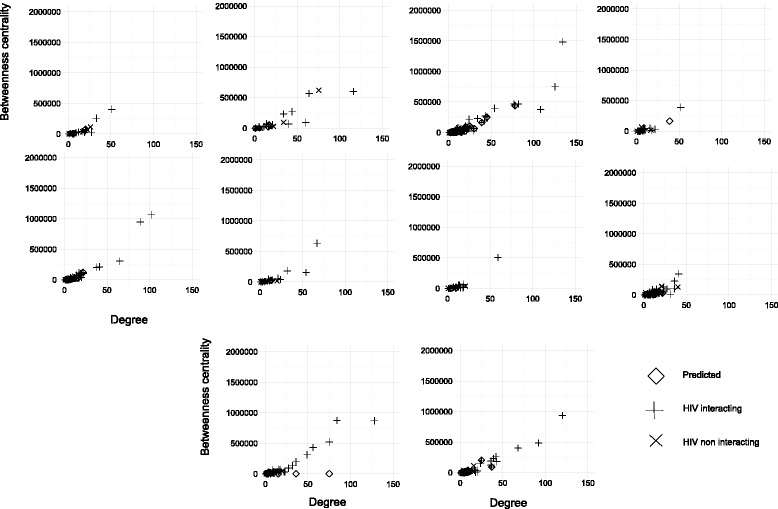
Scatter diagram for 10 selected meta modules showing the degree versus betweenness centrality of HIV-1 interacting proteins, HIV-1 non-interacting proteins, and the proteins that are predicted to interact with HIV-1 proteins. The three categories of proteins are represented by three separate markers

It is established that HIV-1 proteins preferentially attached with highly connected (‘hubs’) and central (‘bottlenecks’) proteins [1]. The proteins having high degree in the interaction network are termed as ‘hubs’ while ‘bottlenecks’ signify high betweenness centrality. Degree of a protein measures the number of protein interacted to it. Betweenness centrality of a node is a measure which counts the number of shortest paths that goes through that node. We have investigated degree and betweenness centrality of proteins involved in the detected meta-modules. This is measured by considering the whole human protein interaction network. The degree and betweenness centrality of HIV-1 interacting and HIV-1 non-interacting proteins involved in the identified meta modules are shown in scatter plot. In Fig. 7 10 scatter diagram are shown for 10 selected modules. All the scatter diagram are provided in supplementary site. In Fig. 7 (a) to (j), X axis represents degree and Y-axis represents betweenness centrality. The red dots signify HIV-1 interacting proteins, and blue dots represent HIV-1 non-interacting proteins. Among the non-interacting sets the proteins, which are predicted to interact with HIV-1 are marked as green dots. From this figure it is evident that the HIV-1 interacting protein set show high degree and betweenness centrality, while non-interacting set show poor values of them. It is noticeable that, among the non-interacting sets the proteins, which are predicted to interact with HIV-1 show high degree and betweenness centrality.

### Predicted interactions

From the meta modules we have predicted 110 interactions between HIV-1 and human proteins. For finding the experimental evidence of our predicted interactions we have extensively searched the existing literature and found some evidence where those predicted interactions are supported. Among them, 26 interactions are found to be well supported by existing literature. Table 2 shows these predicted interactions. All the predicted interactions are provided in the supplementary file. From Table 2, row 1 we noticed that the protein Integrin, Alpha 4 (ITGA4) is predicted to interact with HIV-1 envelop protein env_gp120. In [19] it is reported that HIV-1 surface glycoprotein gp120 binds and signal through integrin Alpha 4 which is also facilitate HIV-1 infection of CD4(+) T cells. In row 2 the protein Myxovirus Resistance (MX2) is predicted to interact with HIV Tat protein. In [20] the MX2 protein is described as an interferon-induced inhibitor of HIV-1 infection. In [21] it is established that HIV-1 protein Tat is interacted with interferon (IFN) stimulated genes (ISG). In row 3 of the Table 2 shows the predicted interaction between HIV-1 tat with Estrogen Receptor Binding Site Associated, Antigen (EBAG9 or RACS1). In [22] it is established that the expression level of apoptosis associated protein RCAS1 (a receptor-binding cancer antigen) is increased by HIV-1 protein Tat. All the 24 predicted interactions are shown in Table 2.

**Table 2:** Predicted interactions supported by existing literature

Sl. No.	HIV-1 protein	human Protein	PUBMED id
1	Env_gp41	ITGA4	PMID: 25008916
2	Tat	MX2	PMID: 24121441
3	Tat	EBAG9	PMID: 17250817
4	Env_gp160	ERGIC3	PMID: 22190034
5	Rev	HNRNPK	PMID: 19808671
6	Rev	SNRPE	PMID: 11780068
7	integrase	SUMO2	PMID: 22895527
8	Gag-matrix	BANF1	PMID: 14645565
9	Rev	HNRNPK	PMID: 19808671
10	Env_gp120	HLA-A	PMID: 1712812
11	Env_gp120	MSN	PMID: 9213396
12	nucleocapsid	TOP1	PMID: 21092135
13	Tat	XBP1	PMID: 10982343
14	Env_gp120	CD63	PMID: 24507450
15	Vpr	CASP8AP2	PMID: 12095993
16	Tat	H2AFZ	PMID: 18226242
17	Tat	SOD1	PMID: 24175971
18	reverse transcriptase	ELAVL1	PMID: 20459669
19	Env_ gp120	LGALS3BP	PMID: 24156545
20	Vpr	PDHA1	PMID: 23874603
21	Env_gp120	MAP2K2	PMID: 15719026
22	Vif	NEDD8	PMID: 23300442
23	Nef	VAMP3	PMID: 20299515
24	Env_gp120	CD69	PMID: 9604776
25	Env_ gp120	HLA-G	PMID: 25472996
26	Tat	SEMA4D	PMID: 22134167

**Fig. 8:**
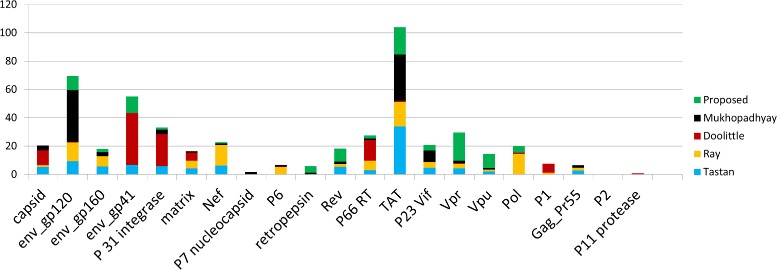
Proportion of predicted interactions involving HIV-1 proteins in five studies

For comparing the predicted interaction with some existing studies we have chosen the predicted interaction set of four literature: Tastan et al. [2], Mukhopadhyay et al., [6], Doolittle et al., [5] and Ray et al., [7]. We perform a study to show the over-representation of HIV-1 proteins in the predicted interaction set in each of the five studies. In Fig. 8 we notice that in most of the predicted interaction sets, HIV-1 protein TAT is significantly over-represented. The possible reason behind that is its essentiality for efficient transcription of the viral genome.

### Assessment of the predicted interaction set

To assesses the predictive performance of our methodology we have performed an analysis to measure sensitivity of the predicted set. Due to the unavailability of true negative set it is not possible to derive specificity of the predicted interactions. Among the selected 2828 proteins 875 HIV interacting proteins are randomly divided into 10 equal-sized subsets (*S*_*i*_). Each subset is considered to be non-interacting for the purpose of this analysis. Using the detected meta modules (over the full set of 2828 proteins) and the proposed prediction method, predictions are made for the proteins in each of these subsets. Note that those that are predicted to be interacting may be considered as true positives, referred to as *p*_*i*_, *i*=1,…,10. Thereafter, the correct prediction for all the subsets is summed up to obtain the total correct predictions. The sensitivity is then defined as $\frac {\sum _{i=1}^{10} p_{i}}{875}$. This entire process is repeated 400 times, and the average sensitivity is computed. We obtained an average sensitivity of 74.77 % by the proposed methodology.

**Fig. 9:**
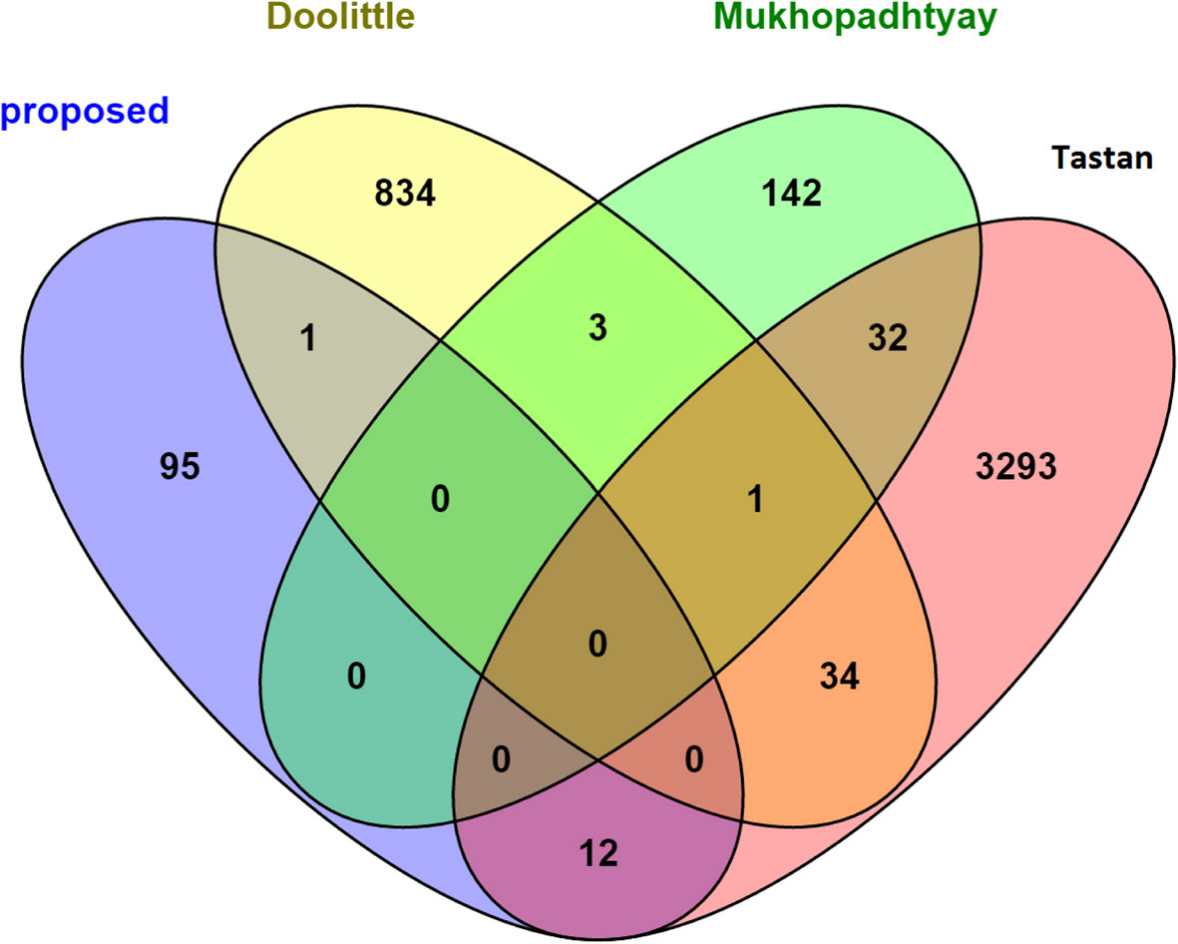
Overlap of the predicted interaction sets of four literatures

To compare the predicted sets of the proposed method with some other state-of-the-art, we detect overlap among the predicted interaction sets of four studies Tastan et al. [2], Mukhopadhyay et al., [6], Doolittle et al., and the proposed method. Figure 9 shows the Venn diagram of the predicted interaction sets. It can be seen from the figure that there is a disagreement among the predicted sets of interactions. Our present study has overlap of 12 interactions with Tastan et al., and one interaction is common with Doolottle et al., but we do not find any interaction common with Mukhopadhyay et al. The possible reason behind this is that the methodologies used for prediction are strongly uncorrelated with each other. Other literature like Bandyopadhyay et al., [1] and Mukhopadhyay et al., [6] support the same fact.

**Fig. 10:**
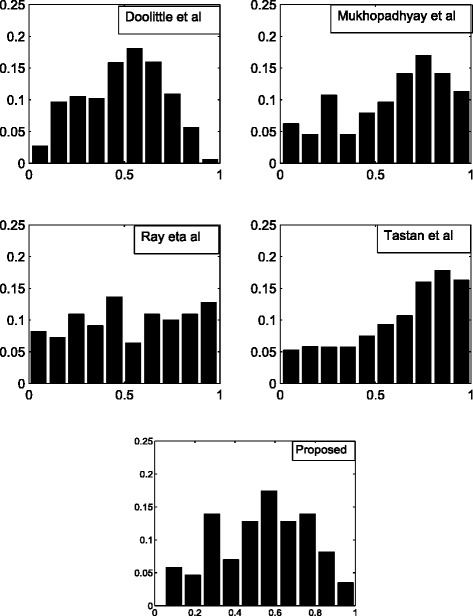
Bar diagrams that show the distribution of interactions with p-values of the five predicted interaction sets. In each plot X-axis represents p-values while Y-axis represents proportion of interactions

To get an overview of the quality of the predicted interaction sets provided by the different approaches, we follow the strategy proposed in Bandyopadhyay et al. [1]. We use conformal prediction approach [23] to assign a confidence level to each of the predicted pairs of each predicted interaction set. Although it is not possible to draw any conclusion about the superiority of the methodologies, still we use this to get an overview of the possible occurrence of the interactions. For assigning confidence to each interaction, conformal prediction approach uses 35 features collected from Tastan et al, [2]. Here, the nonconformity measure is defined on this feature set and a p-value is assigned to each interaction to measure the probability of its occurrence with respect to a previously defined 1063 pairs of interactions from NIAID [8]. Note that the p-values signify the probability of occurrence of an interaction. In Fig. 10 we plot a bar diagram to show the distribution of interactions with p-values of the five predicted interaction sets. From the figure it can seen that over 41 % of interactions of the proposed method have p-value greater than 0.6, where as for Tastan et al. and Doolittle et al. it is over 60 % and for Ray et al. and Mukhopadhyay et al. it is 33.05 and 56.50 %, respectively. A possible reason behind the good performance of Tastan et al. and Doolittle et al. may be that the feature set used by conformal prediction approach is the one collected from Tastan et al.

### GO term and KEGG pathway analysis of meta modules

Gene Ontology and pathway based analysis are the most important and powerful methods for better understanding the biological meaning of the observed expression change. In Table 3 we provide most significant GO-terms, GO-id and KEGG pathway for each of the identified meta module. For this purpose, we have utilized the Database for Annotation, Visualization and Integrated Discovery (DAVID) [14, 24]. In Table 3, row 1, the meta-module consists of 219 proteins, associate with GO term translational elongation (1.5e-15) and KEGG pathway Ribosome (6.7e-16). Translation elongation is a process of successive addition of amino acid residues to a ppolypeptide chain during protein biosynthesis. Elongation factor 1-alpha (EF1alpha) is an essential component of this translation machinery which delivers aminoacyl-tRNA to ribosomes. In [25] it is stated that elongation factor 1-alpha (EF1alpha) binds with the entire HIV-1 Gag polyprotein and inhibits the translation process. It can be noticed in Table 3, row 18 that, the module consisting of 133 proteins are associated with GO term regulation of apoptosis (2.7E-5) and KEGG pathway T cell receptor signaling pathway (1.2E-2). There are many evidences exist that show the connection of HIV-1 with cell apoptosis [26, 27]. Also in [28] it is established that HIV-1 proteins are responsible to alter the T-cell signaling pathways by activating multiple transcription factors. A careful observation in Table 3 reveals that some modules are enriched with different neurodegenerative disease pathways, like Parkinson’s disease (6.9E-4), Huntington’s disease (2.1E-2). In [29] it is demonstrated that HIV-infected peripheral blood mononuclear cells (PBMCs) show overrepresentation in neurodegenerative pathways.

**Table 3:** GO and pathway enrichment of predicted meta modules

Sl. No	No of genes	GO terms	KEGG pathway
1	219	translational elongation (1.5e-15)	Ribosome (6.7e-16)
2	35	positive regulation of transcription, DNA-dependent (2.8e-2)	not found
3	248	RNA processing (7.0E-8)	Ribosome (3.6e-4)
4	29	positive regulation of protein metabolic process (1.1e-3)	Proteasome (6.1e-3)
5	205	translational elongation (1.3E-28)	Ribosome (3.8E-21)
6	32	protein kinase cascade (5.1E-3)	Notch signaling pathway (9.7E-2)
7	31	regulation of actin filament polymerization (5.5E-3)	Cell cycle (5.7E-2)
8	138	regulation of programmed cell death (1.5E-5)	Natural killer cell mediated cytotoxicity (5.1E-4)
9	133	immune response (5.2E-8)	Allograft rejection (1.1E-5)
10	106	cell cycle (7.4E-6)	DNA replication (2.8E-4)
11	92	translational elongation (1.3E-21)	Ribosome (1.1E-19)
12	89	translational elongation (2.4E-15)	Ribosome (2.1E-13)
13	80	RNA splicing (3.1E-13)	Spliceosome (3.1E-5)
14	82	immune response (3.2E-3)	Regulation of actin cytoskeleton (4.9E-3)
15	69	chromatin modification (1.8E-2)	Cell cycle (2.2E-2)
16	41	regulation of cellular protein metabolic process (1.8E-4)	Huntington’s disease (2.1E-2)
17	76	electron transport chain (8.9E-5)	Parkinson’s disease (4.5E-9)
18	68	regulation of apoptotic process (2.7E-5)	T cell receptor signaling pathway (1.2E-2)
19	66	DNA metabolic process (1.9E-3)	Cell cycle (9.3E-2)
20	40	negative regulation of molecular function (1.9E-4)	not found
21	72	regulation of organelle organization (2.2E-3)	Fc gamma R-mediated phagocytosis (1.6E-2)
22	64	RNA splicing (2.9E-10)	Spliceosome (3.9E-9)
23	62	muscarinic acetylcholine receptor signaling pathway (7.5E-4)	Chemokine signaling pathway (1.4E-2)
24	42	RNA processing (7.5E-3)	Spliceosome (2.9E-2)
25	68	purine ribonucleoside monophosphate biosynthetic process (1.8E-3)	Ribosome (5.9E-3)
26	24	immune response (3.6E-4)	Aminoacyl-tRNA biosynthesis (7.8E-2)
27	59	regulation of DNA binding (4.0E-3)	Systemic lupus erythematosus (9.6E-3)
28	27	cellular defense response (5.6E-3)	Endocytosis (5.3E-2)
29	183	oxidative phosphorylation (7.9E-5)	Parkinson’s disease (6.9E-4)
30	19	negative regulation of macromolecule metabolic process (5.3E-2)	not found

## Conclusions

In this article, we propose a novel approach based on integration of different biological data sources, for predicting PPIs between HIV-1 and human proteins. Here, the integration is achieved through non negative matrix factorization (NMF) technique. NMF is utilized here to construct meta-modules from two different categories of modules identified using three different types of data sources, viz., protein-protein interaction (PPI), microarray gene expression and gene ontology based data. The identified meta-modules inherit the biological properties of all those data sources. All these data sources are initially converted to respective biological network, in which the edges capture the similarity between proteins/genes. For example in PPI network, the edge signifies interaction, while in coexpression network an edge represent the correlation similarity between two gene expression profile, and for gene ontology based semantic similarity network the edge represents functional similarity between proteins.

We have analyzed the identified meta-modules biologically and also investigated topological properties of its member proteins in the whole PPI network. It is evident from the analysis that in most of the cases the predicted human proteins show high degree and betweenness centrality. As a result these proteins are demonstrated to be a possible candidate for HIV-1 interactions. We have analyzed the GO terms and KEGG pathway that are associated with the meta modules. We have noticed that most of the modules are enriched with HIV-1 specific GO terms and signaling pathways. Different neurodegenerative pathways like Parkinson’s disease and Huntington’s disease are associated with modules.

We observed that HIV interacting protein in meta-modules show high interactions among them. The proposed prediction technique is guided by this observation. Low p-value also suggests that the observation is statistically significant. For validating the predicted interactions different evidence are collected from existing literature. We have extensively searched and find the literature where the predicted interactions are supported. we have predicted 110 interactions from which we found 44 evidences. We have compared the predicted interactions with predicted interaction set of four literature: Tastan et al., Mukhopadhyay et al., Doolittle et al., and Ray et al. All these studies have utilized completely uncorrelated methodologies for predicting interactions. So, it is not possible to compare these methodologies in a competitive manner instead it could be more appropriate to consider them as collaborative in order to capture the full set of possible interactions. The analysis reveals that our predicted set are overrepresented with the interactions with HIV-1 protein Vpr. Most of the existing predicted sets are overrepresented with HIV-1 protein Tat and envelop protein gp120 and gp41. Our predicted set also captures some proportion of it.

The proposed methodology can be utilized for general computational PPI prediction task. In addition to the prediction task, it can be applied to predict modules by aggregating different data sources. This also can easily be extended to other species or a pair of species or to integrate other auxiliary information to form modules. Thus the method has significant potential for intra or inter-species PPI prediction as well as module detection.
